# Time is ticking for TikTok tics: A retrospective follow‐up study in the post‐COVID‐19 isolation era

**DOI:** 10.1002/brb3.3451

**Published:** 2024-03-11

**Authors:** Kinga K. Tomczak, Jennifer Worhach, Michael Rich, Olivia Swearingen Ludolph, Susan Eppling, Georgios Sideridis, Tamar C. Katz

**Affiliations:** ^1^ Tic Disorders and Tourette Syndrome Program, Department of Neurology Boston Children's Hospital Boston Massachusetts USA; ^2^ Clinic for Interactive Media and Internet Disorders (CIMAID) and Digital Wellness Lab, Division of Adolescent/Young Adult Medicine Boston Children's Hospital Boston Massachusetts USA; ^3^ Occupational Therapy Online OTs, and CBIT Therapy Boston Massachusetts USA; ^4^ Biostatistics and Research Design Center, Institutional Centers for Clinical and Translational Research Boston Children's Hospital, Harvard Medical School Boston Massachusetts USA; ^5^ Department of Psychiatry Boston Children's Hospital Boston Massachusetts USA

**Keywords:** functional tic‐like behaviors, functional tics, tic disorders, Tourette syndrome

## Abstract

**Introduction:**

During the COVID‐19 pandemic, an influx of adolescents presented worldwide with acute onset of functional tic‐like behaviors (FTLBs). Our goal was to evaluate psychosocial factors around onset, to elucidate outcomes after pandemic isolation protocols were lifted, and to examine therapy and medication management.

**Methods:**

A retrospective review was performed of 56 patients ages 10–18 years with new‐onset FTLBs seen at Boston Children's Hospital beginning in March 2020. Demographic factors, medical history, and treatment were evaluated. Patient outcomes were determined retrospectively based on the Clinical Global Impression Improvement (CGI‐I) and Severity (CGI‐S) scales from follow‐up visits. CGI‐I scores assessed the progression of FTLBs; CGI‐S assessed overall function.

**Results:**

Ninety‐six percent of patients were female‐assigned at birth with high rates of comorbid anxiety (93%) and depression (71%). Forty‐five percent were gender‐diverse. Based on scales that assessed FTLBs (CGI‐I) and overall functioning (CGI‐S), up to 79% of patients improved independent of comorbid diagnosis or treatment. Evidence‐based tic‐specific treatments were not more effective than other treatments. A subset of patients had improvement in their FTLBs but not in their general functioning and continued to have other psychosomatic presentations.

**Conclusion:**

While many patients’ FTLBs improved, it is critical to remain alert to patients’ overall function and to assess for other functional neurological disorders and mental health concerns. The tendency of FTLBs to improve in this population, independent of treatment, highlights the unique pathophysiology of FTLBs. Future research on contributing psychosocial factors and specific treatment protocols will allow optimal support for these patients.

## 1 INTRODUCTION

Functional tics, also known as functional tic‐like behaviors (FTLBs), are a subcategory of functional neurological disorders (FNDs, also called conversion disorders in the DSM‐5‐TR (American Psychiatric Association, 2022)) and were previously considered relatively rare (Baizabal‐Carvallo & Jankovic, 2014; Demartini et al., 2015; Pringsheim et al., 2023). FNDs are believed to arise from altered brain networks rather than abnormality in brain structures. Individuals with FNDs experience alterations in motor, sensory, or cognitive functions and experience significant distress (Hallett et al., 2022). These disorders lie on the border of neurology and psychiatry and often present a diagnostic and management challenge that warrants a multidisciplinary approach. In recent years, there has been a paradigm shift from thinking of FND as a diagnosis of exclusion to a more current understanding of FND as defined by positive clinical signs that are not consistent with other neurologic disorders (Aybek & Perez, 2022).

When FND symptoms occur in groups, they have been characterized as a mass psychogenic illness (MPI) (Bartholomew & Wessely, 2002). Reports of MPI arise as early as the Middle Ages (Bartholomew, 2001) and include a precedent for FTLBs as recently as 2011 when 19 females and one male at a high school in LeRoy, NY, developed Tourette Syndrome‐like tics (Novella, 2012). MPI occurs when multiple individuals with risk factors are in close contact with each other, such as at a school or summer camp. Risk factors include being adolescent female, having co‐morbid attention deficit hyperactivity disorder (ADHD), anxiety, or depression (Bartholomew et al., 2012).

Following the onset of the COVID‐19 pandemic, adolescent and adult patients (Nilles et al., 2023), mostly without a prior history of tics, presented with acute onset of FTLBs to clinics and emergency departments worldwide (Malaty et al., 2022; Müller‐Vahl et al., 2022). The increase in FTLBs coinciding with the COVID‐19 lockdown was so rapid and widespread that it was described as a “pandemic within a pandemic” (Olvera et al., 2021). The bizarre and abrupt onset of some of the symptoms was worrisome for parents and clinicians unequipped to diagnose and treat FTLBs. In many cases, extensive medical workups were pursued including imaging, lumbar puncture, viral and strep titers, and serum labs. No clear correlation was found with any infectious or inflammatory process, including SARS‐2 Coronavirus.

FTLBs differ from tics or Tourette Syndrome. FTLBs typically occur in rapid succession within minutes and often lack a premonitory urge for a specific tic. Patients may exhibit movements such as punching oneself, hitting family members, repeating random words or phrases, and blurting out obscenities (Pringsheim et al., 2021). Other previously described characteristics of FTLBs include prevalence among adolescent females, absent family history of tics, inability to suppress movements, and the presence of other functional symptoms (Baizabal‐Carvallo & Jankovic, 2014; Pringsheim et al., 2023). These characteristics were fairly consistent across countries (Cavanna et al., 2023a), leading to creation of an international registry of patients (Martino et al., 2023) as well as creation of consensus criteria for diagnosing FTLBs (Pringsheim et al., 2023) by which patients must present with at least two of three major criteria: age of onset later than age 10, rapid evolution of symptoms, and a constellation of phenomenological symptoms including inconsistencies within the tics, tendency toward complex rather than simple tics that often involve pronounced gestures or phrases, and evolution of tics within hours to days. Further minor diagnostic criteria include co‐morbid psychiatric illness or other FND symptoms, which are seen in a very high percentage of patients (Pringsheim et al., 2023).

Treatment for FTLBs remains varied and is not yet standardized. Experts suggest psychoeducation for patients and families to reduce reinforcement of FTLBs as well as lifestyle modifications including exercise, reducing screen time, promoting a healthy diet, and sleep (Malaty et al., 2022). Randomized clinical trials (RCT) and consensus papers support the use of psychotherapy and/or physical therapy for functional movement disorders (Dallocchio et al., 2016; Nielsen et al., 2017, 2015), occupational therapy for FND (Nicholson et al., 2020), and cognitive behavioral therapy for psychogenic nonepileptic seizures, though to date no RCTs have examined these outcomes specifically for FTLBs. Other treatments include pharmacotherapy to target comorbid diagnoses (Aybek & Perez, 2022; Bravo et al., 2013; LaFrance et al., 2014) though there is a paucity of studies evaluating specific medications in children.

Clinicians still do not fully understand what caused the rise in FTLBs beginning in March 2020. Some have suggested that the stress of the pandemic, including social isolation, increased illness‐related anxiety, increased family tension, and loss of extracurricular activities may have contributed (Buts et al., 2022; Martindale & Mink, 2022; Pringsheim & Martino, 2021; Pringsheim et al., 2021). High rates of anxiety and depression in adolescents during COVID‐19 have been documented Which is an important correlation as both are among the most common comorbidities of FNDs (Patron et al., 2022).

An equally striking and possibly related correlation to the abrupt rise in FTLBs was an increase in social media use among adolescents between March 2020 and March 2022. Many patients who experienced new onset FTLBs reported engaging with social media influencers that exhibited tic‐like behaviors on their platforms, in a phenomenon that came to be known as “TikTok tics” (Amorelli et al., 2022; Forsyth, 2021; Heyman et al., 2021; Hull & Parnes, 2021; Müller‐Vahl et al., 2022; Olvera et al., 2021; Pringsheim et al., 2021; Zea Vera et al., 2022). In many cases, these online sites provided social connections during the pandemic via access to online communities of supportive peers struggling with similar symptoms. However, high rates of social disconnection and mental health disorders existed prior to the COVID‐19 pandemic. The abrupt increase in FTLBs in 2020–2022 suggests that the combination of preexisting marginalization and mental health disorders among vulnerable populations, the lockdown that stymied normal adolescent autonomy‐seeking away from family toward peers, and the rapid shift to social interactions occurring online rather than in person created a perfect storm of sociogenic illness risk factors.

As at other pediatric treatment centers, a large influx of patients presented with FTLBs at our tertiary care academic hospital starting in March 2020 with the highest peak occurring in the midst of the pandemic and gradually improving with the return to open society. The goal of this study was to characterize the outcomes of children with FTLBs since COVID‐19 pandemic isolation protocols have been lifted. Secondary goals were to identify predisposing factors that may foster a higher risk of developing FTLBs and to examine treatment interventions.

## 2 MATERIALS AND METHODS

We retrospectively reviewed medical records of patients ages 10–18 seen at Boston Children's Hospital with new onset FTLBs beginning in March 2020. In total, 58 patients were identified as having FTLBs, two were excluded because outcome data were not available, resulting in a cohort of 56 patients. Clinic notes were reviewed, and data were collected regarding symptom onset, previous history of tics, co‐occurring diagnoses, and treatment.

**FIGURE 1:**
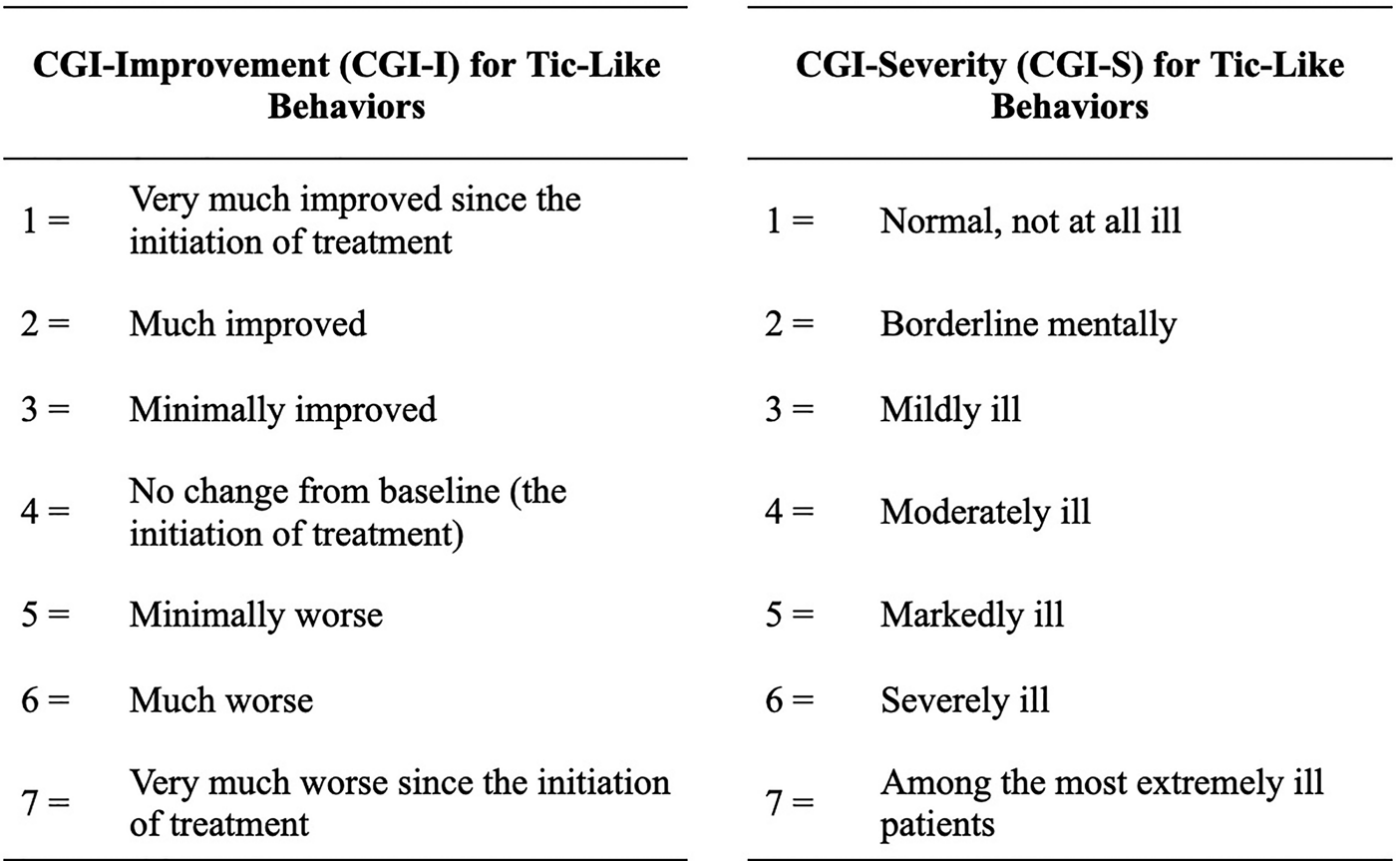
Clinical Global Impression Improvement and Severity Scales.

We retrospectively reviewed results from the Clinical Global Impression Improvement Scale (CGI‐I) and Clinical Global Impression Severity Scale (CGI‐S) at follow‐up visits from August 2022 to January 2023. The average duration of follow‐up was 518 days (min 137, max 894). These scales are used by clinicians to track patient progress over time. CGI‐I scores range from 1 (much improved) to 7 (very much worse) and CGI‐S scores from 1 (Normal, not at all ill) to 7 (among the most extremely ill patients) (Busner & Targum, 2007) (Figure 1).

In our practice, CGI‐I was used specifically to assess progression of FTLBs based on parent reports. Patients with CGI‐I scores of 1–2 were considered to have improved; patients with CGI scores of 3 or higher were categorized as not improved.

Separately, CGI‐S determinations were based on overall functioning, considering factors such as symptom severity of comorbid conditions, and the presence of alternative functional neurological symptoms. Patients with a CGI‐S score of 1–3 were considered to have none or only mild functional impairments. Patients with a score of 4 or higher were categorized as moderately to severely functionally impaired.

This study was approved by the Boston Children's Hospital Institutional Review Board with a full waiver of consent.

### 2.1 Data analyses

Data were analyzed using the chi‐square test, which examines differences in frequency among two or more categorical variables. All the analyses involved one degree of freedom, and the level of significance was set to 5% for a two‐tailed test. Power for the chi‐square test equal to 80% was achieved for a medium effect size (*w* = 0.5) with *n* = 32 participants. This number was sufficient for analyses involving the full sample of *n* = 56 and most analyses using subsets of the data.

## 3 RESULTS

In total, 56 patient records were analyzed with a mean age of symptom onset at 14 years. The majority (68%) did not have a history of tics. Ninety‐two percent (52/56) presented with comorbid anxiety disorders and 71% (40/56) with depressive disorders. Thirty‐eight patients had both diagnoses (70%). ADHD was reported in 45%, obsessive‐compulsive disorder (OCD) in 23%, and autism spectrum disorder in 7%. Patients reported comorbid functional neurologic symptoms such as psychogenic nonepileptic seizures in 38%, dissociative events (23%), paralysis or gait impairment (13%), and psychogenic tremors (7%) in addition to their FTLBs, in some cases with several functional symptoms within the same patient (Table 1).

**TABLE 1:** Demographics (*N* = 56).

	Number/Mean	%/SD
Sex assigned at birth		
Male	2	4%
Female	54	96%
Gender orientation		
Cisgender	31	55%
Gender‐diverse	25	45%
Co‐occurring psychiatric diagnoses (may overlap among participants)		
Anxiety	52	93%
Depression	40	71%
Attention deficit hyperactivity disorder	25	45%
Prior history of tics	18	32%
Obsessive‐compulsive disorder	13	23%
Autism spectrum disorder	4	7%
Co‐occurring functional diagnoses		
Psychogenic non‐epileptic seizures	18	32%
Dissociative events	13	23%
Paralysis or gait impairment	7	13%
Psychogenic tremor	4	7%
Age of onset	14	1.9 SD
Stressor prior to onset	30	54%

*Note*: For continuous variables, the median and SD are displayed. For categorical variables, the count of participants and percentage are displayed.

While most patients were females assigned at birth (96%), 45% of patients identified as either nonbinary or transgender (mostly trans male, with one individual identifying as trans female). Over 53% of FTLB patients reported a major stressor in their life prior to the onset of tics; 25% of the cohort specifically referenced the COVID‐19 pandemic. Others reported stressors such as a recent medical diagnosis, a move, or a death in the family.

Based on clinician‐rated CGI‐I scores, the FTLBs in 79% of patients were “very much improved” or “much improved” at follow‐up visits. Similarly, on the CGI‐S scale used for overall functioning, 71% of patients were rated by their clinician as normal to only mildly ill, while 29% were reported to be moderately to extremely ill at the time of follow‐up (Table 2).

**TABLE 2:** Clinical global impression tic improvement and overall functioning at follow‐up.

	CGI improved		CGI not improved		Chi‐square *p*‐value
	Number	Percentage	Number	Percentage	
Overall CGI‐I Score (tic improvement)	44	79	12	21	<.05
Tic improvement and co‐occurring diagnoses					
ADHD	20	80	5	20	<.01
Anxiety	40	77	12	23	<.001
Depression	31	78	9	23	<.01
Overall CGI‐S Score (overall functioning)	40	71	16	29	<.01

*Note*: The table shows the percentage of patients in our cohort who improved/did not improve on CGI‐I and CGI‐S scales. The CGI‐I scale (used to assess FTLBs) is also broken down by comorbid diagnosis of ADHD, anxiety, and depression. Due to the small sample size, we were not able to distinguish differences in CGI‐I between diagnoses of ASD or OCD. Abbreviations: ADHD, attention deficit hyperactivity disorder; ASD, autism spectrum disorder; CGI‐I, Clinical Global Impression Improvement Scale; CGI‐S, Clinical Global Impression Severity Scale; FTLB, functional tic‐like behavior; OCD, obsessive‐compulsive disorder.

Gender‐diverse patients were significantly more likely to be rated as moderately to extremely ill at follow‐up (*p* < .05) with regard to overall functioning (CGI‐S). However, the improvement of tic‐like behaviors (CGI‐I) did not vary by gender identity (Table 3).

**TABLE 3:** Cisgender and Gender‐diverse Outcome Comparison.

	Total number	CGI Improved		Chi‐square *p*‐value
	Number	Number	Percentage	
CGI‐I (tic improvement)				
Cisgender	31	26	84	.726
Gender‐diverse	25	18	72	
CGI‐S (overall functioning)				
Cisgender	31	26	84	<.05
Gender‐diverse	25	14	56	

*Note*: The table shows the percentage of patients that improved on the CGI‐I and CGI‐S broken down by gender identity. Abbreviations: CGI‐I, Clinical Global Impression Improvement Scale; CGI‐S, Clinical Global Impression Severity Scale;

Eighty‐six percent of patients received some form of mental health care, including cognitive behavioral therapy, dialectical behavioral therapy, art therapy, and other types of behavioral care. For many patients, this therapy was in addition to medication such as selective serotonin reuptake inhibitors (SSRIs), selective serotonin and norepinephrine reuptake inhibitors (SSNRIs), antidepressants, antipsychotics, and alpha agonists. Of the 48 patients who engaged with mental health care, 19 also received cognitive behavioral intervention for tics (CBIT), a first‐line evidence‐based intervention for tic disorders that involve (a) awareness of the involuntary nature of tics, (b) detection of premonitory urges, (c) environmental and activity modification, (d) emotional support, and (e) competing responses. Seventy‐nine percent of patients had improvement in FTLBs, regardless of the type of therapy employed. CBIT did not confer any improvement in treatment outcomes above mental health therapy alone.

Ninety‐one percent of patients were treated with medication, and 82% of patients received an SSRI/SSNRI at some point during their treatment. Of those treated with an SSRI/SSNRI, 76% had improvement in their FTLBs. Since the majority of patients were on medication and participated in mental health therapy, we were unable to compare the results of therapy alone versus medication alone. However, we did analyze patients who received mental health therapy but were not treated with SSRIs/SSNRIs and found that 78% improved. Thus, patients in all treatment groups showed similar improvement in FTLBs (Table 4).

**TABLE 4:** Treatment outcome.

CGI outcome	Number of subjects	Number of CGI improved	Percentage	Chi‐square *p*‐value
Therapy				
Mental health therapy (with CBIT)	19	15	79	<.05
Mental health therapy (without CBIT)	29	23	79	<.01
Medications				
Mental health therapy with no SSRI/SSNRI	9	7	79	<.05
SSRIs/SSNRIs	46	35	76	<.001
Multiple medications^a^	51	40	78	<.001

*Note*: The table shows the number of subjects improved based on treatment. Abbreviations: CBIT, cognitive behavioral intervention for tics; CGI, clinical global impression; SSNRIs, selective serotonin and norepinephrine reuptake inhibitor; SSRIs; selective serotonin reuptake inhibitors.

^a^ Multiple medications include SSRIs/SSNRIs, antianxiety, antidepressants, antipsychotics, and alpha agonists.

## 4 DISCUSSION

**FIGURE 2:**
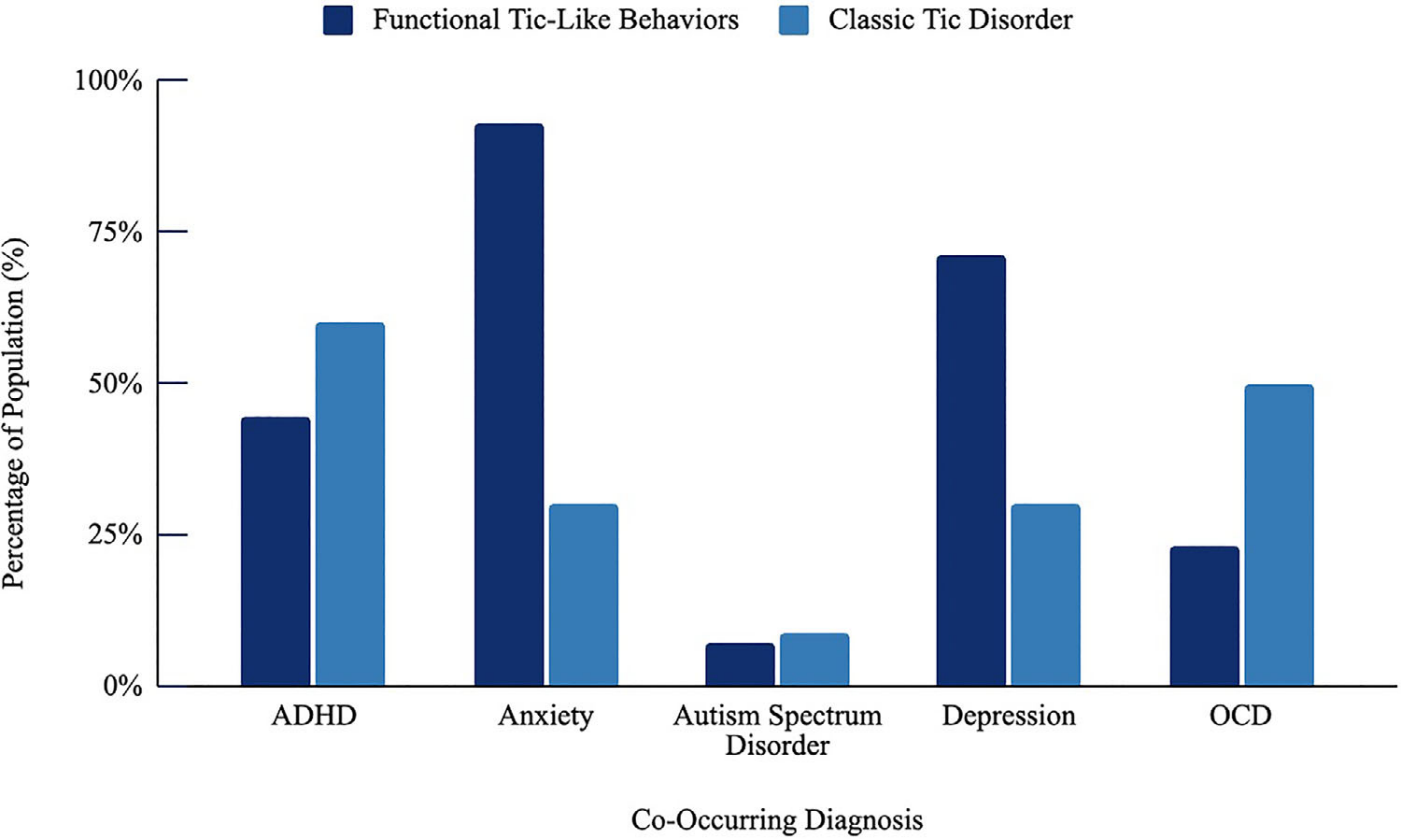
Co‐occurring diagnoses in patients with tics versus FTLBs. Displays the percentage of children and adolescents with FTLBs versus tics who also have comorbid ADHD, anxiety, ASD, depression, and/or OCD. Dark blue represents FTLBs, light blue is tics. Compared to adolescents with tic disorder, our cohort reported having higher rates of co‐morbid anxiety (93% vs. 30%) and depression (71% vs. 30%). Presentation of co‐morbid OCD and ADHD, in contrast, is more common in adolescents with tic disorders than FTLBs (OCD = 50% vs. 23%; ADHD = 60% vs. 45%). In our cohort, the percentage of patients with FTLBs and ASD is similar to those with classic tics (9% vs. 7%), though given our small cohort size, this remains to be validated in a larger sample. *Data from*: Termine C et al., (Termine et al., 2006) Yan J et al., (Yan et al., 2022) Hirschtritt ME et al., (Hirschtritt et al., 2015) Ogundele MO et al., (Ogundele & Ayyash, 2018) and Gulisano M, Barone R, Mosa MR, et al. Incidence of Autism Spectrum Disorder in Youths Affected by Gilles de la Tourette Syndrome Based on Data from a Large Single Italian Clinical Cohort. *Brain Sci*. 2020;10. ADHD, attention deficit hyperactivity disorder; ASD, autism spectrum disorder; FTLB, functional tic‐like behavior; OCD, obsessive‐compulsive disorder.

A surge in FTLBs presented to Boston Children's Hospital during the COVID‐19 pandemic, similar to trends seen worldwide. The high prevalence of females (96%), as well as the mean age of functional tic onset at 14, differ considerably from tic disorders which tend to present more commonly in males prior to puberty, often as early as 4–5 years of age. Consistent with what is known about FND, over 92% of this cohort had a comorbid diagnosis of anxiety and over 70% had a diagnosis of depression. This is in stark contrast to adolescents with tic disorders, among whom rates of anxiety or depression are estimated to be approximately 30% (Hirschtritt et al., 2015) (Figure 2). Current theories suggest that FND arises from networks in the brain that overlap with anxious and depressive disorder symptomatology and that many FND symptoms are essentially somatization of anxiety or depression‐spectrum pathology. More specifically, current theories postulate that FND is driven by impairments in self‐awareness and predictive coding, which are neurobiologically affected by stress, uncertainty, and neurally encoded beliefs, all of which are highly affected by mood and anxiety disorders (Milano et al., 2023). Our findings are similar to other studies of this population, which also demonstrated mean age of onset around 14–15, high rates of anxiety and depression, rapid progression of symptoms, and higher than expected prevalence of FND symptoms (Cavanna et al., 2023a; Prato et al., 2022; Pringsheim et al., 2023).

Further differentiating FTLB sufferers from those with tics is that fewer than ¼ of our patients had comorbid OCD, in contrast to tic disorders in which the comorbidity with OCD is estimated at 40–50% (Hirschtritt et al., 2015; Termine et al., 2006; Yan et al., 2022). This discrepancy is consistent with the largest controlled study to date which found that overall a lack of tic‐related OCD behaviors and lack of family history were the strongest predictors of functional tics versus neurodevelopmental tics (Cavanna et al., 2023b).

Less than half (45%) of patients in our cohort had ADHD, while it is estimated that 60% of patients with tic disorders have ADHD (Ogundele & Ayyash, 2018). This discrepancy may be related in part to the fact that ADHD occurs more commonly in latency‐aged males (Willcutt, 2012), while functional tics occur overwhelmingly in adolescent females (Pringsheim & Martino, 2021; Pringsheim et al., 2021). We found a 7% prevalence of autism spectrum disorder (ASD). While the size of this cohort was too small to draw statistical conclusions, many of the FTLB patients, even those without a formal ASD diagnosis, reported long‐standing difficulties with social pragmatics and maintaining relationships.

### 4.1 Outcomes

The CGI data gathered at follow‐up visits from August 2022 to January 2023 showed that approximately three‐fourths of patients had improvement in FTLBs, independent of comorbid diagnoses. This is in keeping with findings across other sites (Howlett et al., 2022; Nilles et al., 2023; Prato et al., 2022) and in contrast to tic disorders which tend to be more treatment‐resistant. The cessation of COVID‐19 isolation protocols likely reduced the burden of depression and anxiety. Increased opportunities for in‐person socialization decreased the use of social media sites with influencer validation of tic‐like behaviors and increased peer pressure against impairing and socially unacceptable behaviors.

Interestingly, the largest discrepancy in improvement was between patients who identify as gender‐diverse compared to patients who identify as cisgender. Both groups had similar improvement in their FTLB‐specific CGI‐I scores, but gender‐diverse patients demonstrated less improvement in CGI‐S scores of overall functioning. This population remains vulnerable to mental health struggles and likely remains at higher risk of sociogenic illnesses.

Clinically, we observed that symptom improvement and overall functioning were stratified into three groups, a finding that warrants further statistical quantification in a larger cohort:

*Group 1*: Patients’ tics improved (CGI‐I), overall functioning improved (CGI‐S), and there were no new onset neuropsychiatric symptoms. High rates of improvement were seen regardless of co‐morbid diagnosis or treatment modality. This is consistent with other studies that have found spontaneous remission rates as high as 20% without treatment (Martino et al., 2023).

*Group 2*: Patients’ tics improved, although their overall functioning and mental health did not. For many, FTLBs evolved into other functional neuropsychiatric symptoms. This subset of patients continues to seek primary and specialized care.

*Group 3*: Patient's FTLBs are unchanged, and their overall functioning remains poor. Many of these patients seem unmotivated to improve as they identify with their tics and associated supportive communities.

Among our study cohort, 48 (86%) patients pursued psychotherapy, with 34% also receiving CBIT. While other studies have examined the role of psychotherapy for anxiety/depression, such as cognitive behavioral therapy (CBT) or CBIT in treating FTLBs (Nilles et al., 2023), our study included a larger cohort of patients receiving CBIT for functional tics. We found that CBIT conferred no additional benefit. The effectiveness of CBIT for FTLBs was hindered in some patients by extreme emotional dysregulation, co‐occurrence of multiple tic‐like symptoms at the same time, or lack of access to CBIT providers. Many patients enjoyed the sense of inclusion and attention (often on social media) that accompanied their FTLB diagnosis and were reticent about treatment as compared to patients with tics for whom the tics are socially embarrassing.

Forty‐six (82%) of our patients were prescribed an SSRI or SSNRI during their treatment; of those on an SSRI or SSNRI, 76% (35/46) improved. This rate of improvement is similar to patients who received therapy without an SSRI or SSNRI (78%). This finding is consistent with well‐studied outcomes for anxiety and depression that have shown that treatment with therapy alone is equally effective compared to treatment with SSRIs at 6‐month follow‐up (March et al., 2007), Other studies have also found that the CBT and SSRIs were effective for promoting symptom improvement (Howlett et al., 2022; Nilles et al., 2023).

Fifty‐one patients (91%) underwent multiple medication trials from multiple classes, including not only SSRIs/SSNRIs but also alpha agonists, stimulants, and/or antipsychotics most commonly, and mood stabilizers, topiramate, gabapentin, bupropion, or mirtazapine less commonly. Medication administration may have been serial or concurrent depending on the patient and regardless of the combinations used, 78% of patients improved. Given the diversity of medications used, we were unable to statistically evaluate specific medication combinations.

The widespread improvement among these patients regardless of treatment plan suggests that improvement in FTLBs correlated less with the specific treatment and more with the reopening of society and resolution of co‐morbid symptoms for most patients. Alternatively, overlapping medications from multiple classes may have served to treat comorbid anxiety, depression, OCD, or ADHD or provided a placebo effect which led to overall improvement in functioning and FTLBs. Given our limited sample size, further study is required for the development of medication protocols for treating FTLBs.

### 4.2 What have we learned?

The complex etiology of the abrupt onset and later improvement of FTLBs during the COVID‐19 pandemic remains unclear. Several studies have discussed potential contributing factors (Buts et al., 2022; Heyman et al., 2021; Martindale & Mink, 2022; Pringsheim & Martino, 2021; Pringsheim et al., 2021) and made excellent suggestions for management (Malaty et al., 2022), emphasizing the role of psychoeducation in FND treatment, reduction of known triggers such as social media, and discouragement of positive reinforcement. However, given the acuity seen in our clinics, second‐ and third‐line treatment with therapy or medication was often indicated although large‐scale studies of specific medication protocols are lacking. Even experienced neurologists and psychiatrists relied on intuition as to what class of medication to initiate or when to add an alternative.

Similarly, no clear protocols exist for behavioral therapy targeting FTLBs. Classic CBIT protocols were less effective for FTLBs in our study group, which may be due to a lack of willingness to embrace treatment but may also be due to variability between CBIT providers who were unsure how to modify the protocol to address FTLBs. A new modified CBIT protocol that combines urge awareness drawn from CBIT, exposure response prevention, sensory grounding drawn from OT, and acceptance of emotions and somatic symptoms as done in CBT is now being proposed (Maxwell et al., 2023). Buy‐in from parents and patients is essential.

The aftermath of the COVID‐19 pandemic increased awareness of mental health struggles and neurodiversity. While this is a positive advancement, it may be paradoxically complex for patients with FTLBs. From our experience, some patients found validation and identity in their diagnoses or identified with Tourette Syndrome even if they did not meet the criteria. Patients came to feel part of an online identity‐based community at a time when in‐person interactions were limited. This can present serious challenges: identity reinforcement drives FTLBs to persist which may interfere with functioning. Furthermore, it may result in ostracism by neurotypical peers, further reinforcing involvement in FTLB communities at the expense of in‐person connections. Parents and clinicians must carefully balance celebrating neurodiversity without enhancing vulnerability among children who are susceptible.

Though FTLBs largely resolved, the underlying psychosocial predisposing factors including social disconnection, academic pressure, or other mental health disorders did not. A number of our patients experienced evolution of their FND symptoms even after FTLBs resolved. Some patients progressed to psychogenic non‐epileptic seizures, functional abdominal pain, functional paralysis or muscle weakness, eating disorders, or, in extremecases, self‐harm. Pediatricians must remain alert to the fragility of these patients and their predisposition to functional presentations. This phenomenon was especially evident among gender nonconforming patients, who are arguably more vulnerable than cis‐gender peers.

The tight communities, such as schools, convents, and small towns in which FND historically occurred were reprised in 2020–2022 in a social media community that extended around the world. We now recognize how effectively social media has allowed isolated young people to form virtual bonds that can lead to both healthy and unhealthy conditions.

Limitations of this study include that this is a retrospective chart review at a single site which resulted in a relatively small sample size of participants with this rare condition. Researchers were not blinded when assessing CGI scales. Nevertheless, our data and clinical experience draw attention to the lack of targeted protocols for the treatment of FTLBs.

## 5 CONCLUSION

The gap in clinical awareness of FTLBs is problematic as patients are at risk for FND generally, and, as we observed, may progress to other functional symptoms. Clinicians must be able to recognize and treat FNDs to avoid diagnostic confusion and stigmatization of patients. Large‐scale studies are warranted to develop diagnostic strategies. Treatment protocols are needed, ideally through creation of FND‐focused clinics that offer a multidisciplinary approach including psychopharmacology, psychological therapy, CBIT, occupational therapy, physical therapy, and family psychoeducation as well as coordination between providers.

## AUTHOR CONTRIBUTIONS

**Kinga K. Tomczak**: Conceptualization; writing—original draft; investigation; writing—review and editing; supervision; methodology; project administration. **Jennifer Worhach**: Conceptualization; visualization; writing—review and editing; writing—original draft; data curation; project administration; methodology. **Michael Rich**: Writing—review and editing; writing—original draft. **Olivia Swearingen Ludolph**: Writing—review and editing; visualization. **Susan Eppling**: Writing—review and editing. **Georgios Sideridis**: Formal analysis; writing—review and editing; validation; methodology. **Tamar C. Katz**: Conceptualization; writing—review and editing; investigation; supervision; writing—original draft; methodology.

## CONFLICT OF INTEREST STATEMENT

The authors have indicated they have no potential conflicts of interest to disclose.

### PEER REVIEW

The peer review history for this article is available at https://publons.com/publon/10.1002/brb3.3451.

## Data Availability

The data that support the findings of this study are available from the corresponding author upon reasonable request.
